# Promising candidates from drug clinical trials: Implications for clinical treatment of Alzheimer's disease in China

**DOI:** 10.3389/fneur.2022.1034243

**Published:** 2022-11-15

**Authors:** Yuxia Cao, Feng Yu, Yi Lyu, Xianfu Lu

**Affiliations:** ^1^School of Basic Medicine and Clinical Pharmacy, China Pharmaceutical University, Nanjing, China; ^2^Department of Anesthesiology, Minhang Hospital, Fudan University, Shanghai, China; ^3^Department of Anesthesiology (High-Tech Branch), The First Affiliated Hospital of Anhui Medical University, Hefei, China; ^4^Department of Anesthesiology, Anqing First People's Hospital of Anhui Medical University, Anqing, China

**Keywords:** Alzheimer's disease, clinical trials, drug development, clinical treatment, China

## Abstract

Alzheimer's disease is the most common neurodegenerative disease. Prior to 2017, National Medical Products Administration approved only four drugs to treat Alzheimer's disease, including three cholinesterase inhibitors and one N-methyl-D-aspartate receptor antagonist. We queried ClinicalTrials.gov to better understand Alzheimer's drug development over the past 5 years and found 16 promising candidates that have entered late-stage trials and analyzed their impact on clinical treatment of Alzheimer's disease in China. The 16 compounds selected include disease-modifying therapies and symptomatic therapies. The research and development pipeline now focuses on disease-modifying therapies such as gantenerumab, aducanumab, ALZ-801, ALZT-OP1, donanemab, lecanemab, simufilam, NE3107, semaglutide, and GV-971, which could put an end to the situation where Alzheimer's patients in China have no effective treatment alternatives. The reuse of drugs or combinations currently under investigation for the psychiatric treatment of Alzheimer's disease, including AXS-05, AVP-786, nabilone, brexpiprazole, methylphenidate, and pimavanserin, could provide physicians with additional treatment options. Although most of these drugs have not been explored in China yet, due to the current development trend in this field in China, it is expected that China will be involved in research on these drugs in the future.

## Introduction

Alzheimer's disease (AD) is a form of dementia that affects memory, thinking, and behavior. It is the most common disease among neurodegenerative disorders. Symptoms include cognitive dysfunction, mental problems, behavioral disturbances, and difficulty performing activities of daily living, all of which have a significant impact on patients' daily lives (1). As the world's population continues to age, the incidence of AD is increasing. According to the 2019 Global Burden of Disease Study, AD is the leading cause of death in people over 75 years of age (2). Incidence rates in people over 65 and 85 are 5% and more than 30%, respectively (3). There are an estimated 50 million worldwide, including 10 million in China. It is estimated that there will be about 130 million Alzheimer's patients worldwide by 2050 (3).

In the Chinese Guidelines for the Diagnosis and Treatment of Alzheimer's Disease published in 2020 (4), there are mainly two types of drugs approved to alleviate cognitive impairment in AD patients, including cholinesterase inhibitors (donepezil, rivastigmine, and galantamine) and glutamate receptor antagonists (memantine), as well as drugs to treat psychiatric symptoms in AD patients. All of the above medications treat symptoms only and have limited effects on AD patients. These medications are far from meeting the needs of the current medical system in light of the increasing prevalence of AD. As research found, amyloid-beta (Aβ) accumulation, the growth of neurofibrillary tangles (NFT), and tau protein hyperphosphorylation are the major causes of AD (5). Increased accumulation of reactive oxygen species (ROS) induces oxidative stress and neuronal cell abnormalities which is one of the pathogenesis of AD (5). Anti-Aβ drugs, anti-tau drugs, and antioxidants, among others, may become promising preventive and therapeutic species for AD. However, these drugs are still under active development, and future research results are unknown.

The development of novel drugs has increased dramatically due to the enormous and largely unmet clinical need. However, failures in this area of drug development are common. According to reliable sources, the failure rate for cancer drug development is 92%, while the failure rate for AD clinical trial development can be as high as 99.6% (6). It can be argued that pharmacological research and development (R&D) in the field of AD treatment is slow compared to other disease areas and is in a state of constant wandering and investigation. Research progress on potentially effective neuroprotectors and therapeutic strategies that prevent the development of AD is stalled because of a lack of adequate understanding of the intricate mechanisms of neurodegeneration (7). Apart from the above reasons, imperfect preclinical models and lack of validated diagnosis contribute to the high failure rate in the development of AD (8). The huge demand for AD drugs and the high failure rate in R&D require us to capture the latest AD candidates in late-stage development and then explore their potential impact on the treatment of AD patients in China in the future. In this paper, we conduct an annual review of the AD drug development pipeline and present the results of our 2017–2021 pipeline analysis as presented on ClinicalTrials.gov.

**Figure 1:**
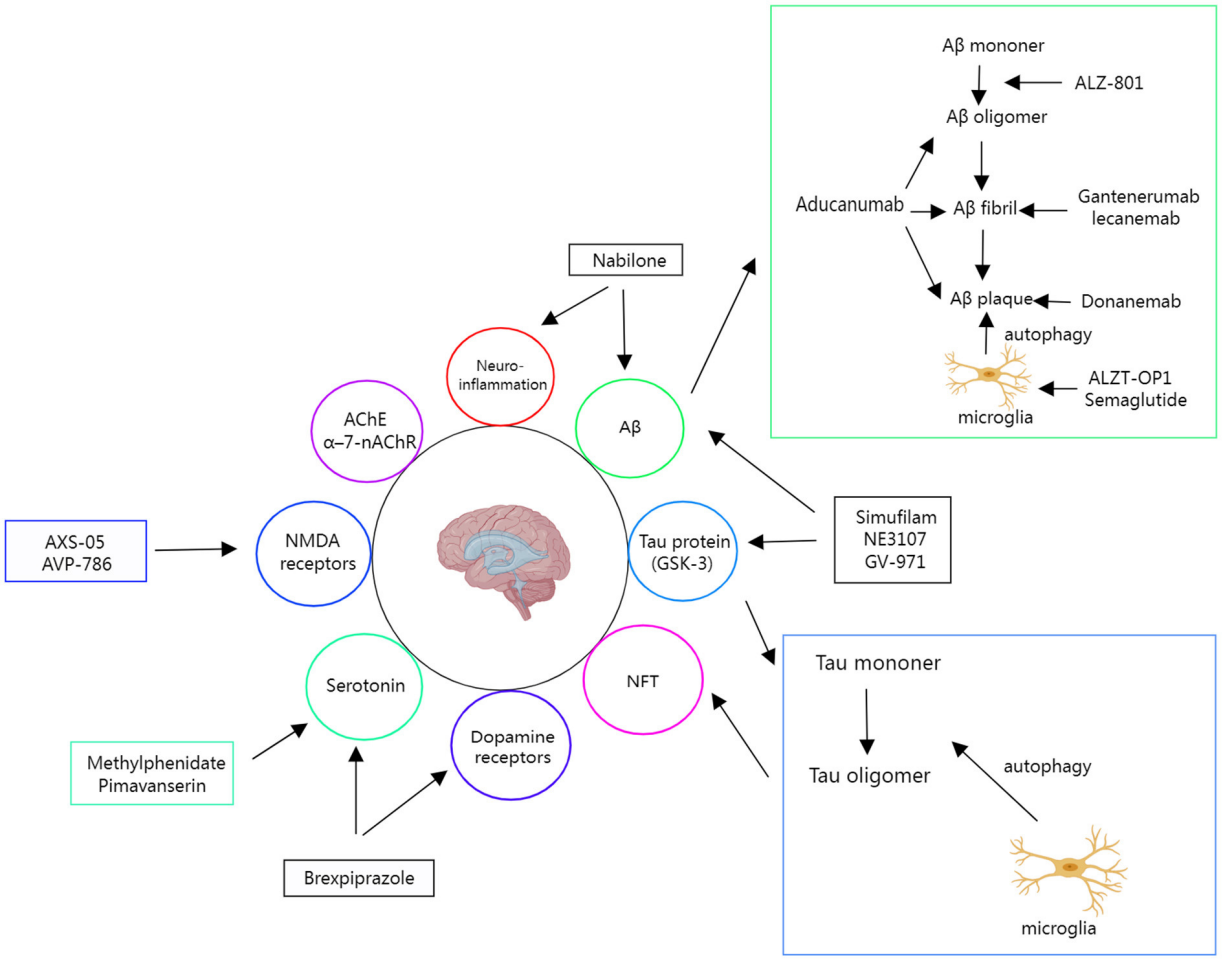
Various therapeutic targets in AD and candidates entering late-stage investigations.

A total of 473 studies were included in our database. These studies are mainly conducted in the United States (US), Europe, China, and other increasingly aging regions. Phase I and Phase II studies accounted for a substantial proportion of the total, 36.36 and 41.86%, respectively. From these studies, we selected 16 compounds that are in late-stage development. Figure 1 illustrates various therapeutic targets in AD.

## Disease-modifying therapy

### Anti-amyloid strategies

#### Aducanumab

Aducanumab, marketed as aduhelm, is an Aβ-direct antibody manufactured by Biogen. Treatment with aducanumab should be initiated in patients with mild cognitive impairment (MCI) or mild dementia (9). It is suitable for all genotypes of early AD (10). The chemical structure of aducanumab is not available online, but studies have found that it binds to the N-terminus of Aβ in an extended conformation (11). Its mechanism of action (MOA) involves binding of aggregated forms of Aβ rather than monomers. Aβ may contribute to cell death and tissue loss in parts of the brain critical for memory, thinking, learning, and behavior. In a first-in-human study (NCT01397539), a dose of 30 mg/kg demonstrated an acceptable safety and tolerability profile, although patients receiving 60 mg/kg of aducanumab experienced serious adverse events (SAEs) and symptomatic amyloid-related imaging abnormalities (ARIA) that resolved at weeks 8–15 (12). Biogen announced in December 2014 that the drug had entered Phase III. However, ARIA-edema (ARIA-E) and ARIA-hemorrhage (ARIA-H), two of the most common adverse events (AEs), occurred in 362 of the 1,029 patients (35.2%) in the 10 mg/kg group in the phase 3 randomized clinical trials (RCTs) of aducanumab EMERGE (NCT02484547) and ENGAGE (NCT02477800) (13). In March 2019, Biogen and Eisai announced that all ongoing aducanumab trials would be discontinued due to the likelihood that EMERGE and ENGAGE would miss their primary endpoints in the interim analysis. Fortunately, Biogen later announced that EMERGE had met its primary endpoint. Key endpoints were significantly reduced in participants who received a high dose, and secondary endpoints declined less. Although patient progress was delayed in the low-dose group, the differences were not statistically significant compared with the placebo group. Although the primary objective of the ENGAGE study was not met, an exploratory analysis showed that patients taking 10 or more mg/kg deteriorated more slowly (14). Based on the results of the above studies, aducanumab may produce a dose-dependent reduction in amyloid and some reduction in phospho-tau (p-tau) in the cerebrospinal fluid (CSF). The Food and Drug Administration (FDA) granted fast-track approval for aducanumab on the condition that Biogen complete a post-approval clinical trial in August 2021 to confirm the drug's efficacy (15).

#### ALZ-801

**Figure 2:**
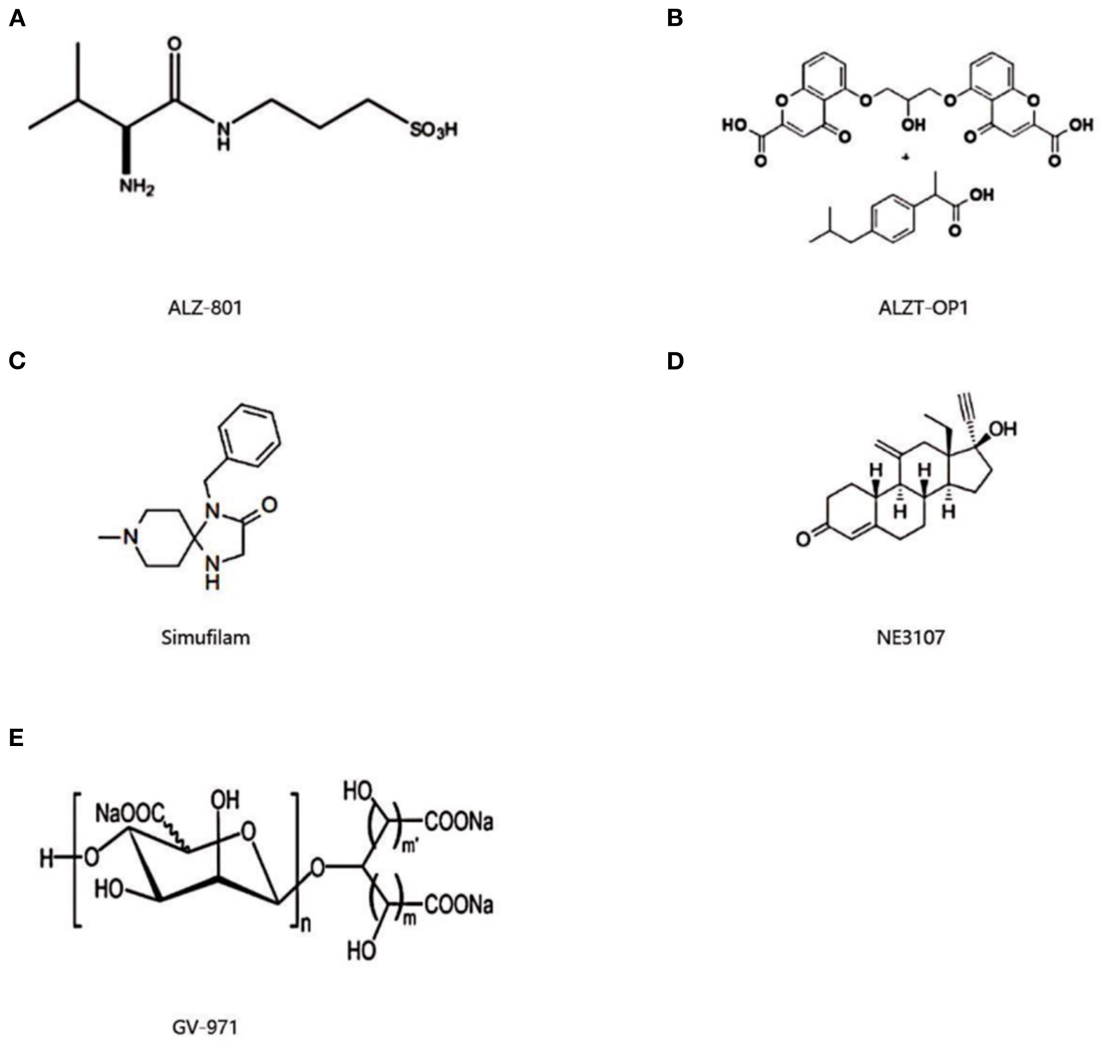
Chemical structure of ALZ-801 **(A)**, ALZT-OP1 **(B)**, simufilam **(C)**, NE3107 **(D)**, and GV-971 **(E)**.

Alzheon developed ALZ-801 (Figure 2A). Several ALZ-801 molecules surround and engage with Aβ monomers using an enveloping MOA, maintaining their structure and preventing the synthesis of neurotoxic oligomers (16). This prevents the hippocampus' shrinkage, which is linked to the onset and progression of the disease (16). ALZ-801 is an orally accessible valine-conjugated tramiprosate prodrug (17). It and tramiprosate are both converted to 3-sulfo-propanic acid (3-SPA), which is present in the brain and inhibits Aβ42 aggregation (17). Compared to tramiprosate, ALZ-801 has better gastrointestinal tolerability and more stable plasma levels, resulting in higher brain penetration (18). Patients with moderate AD and APOE4/4 homozygotes are the target population (19). Alzheon began a phase II open-label biomarker study in September 2020 (NCT04693520) with results expected in July 2023. Patients with early AD and one or two copies of APOE4 are enrolled in the study. Alzheon presented interim results in February 2022 on 80 patients treated for 6 months, reporting a 29% decrease in plasma P-tau18 from baseline, a similar decrease in the p-tau181/Aβ42 ratio, and improvement in the Rey Auditory Verbal Learning Test (RAVLT) (20). From May 2021 to April 2024, the phase 3 trial (NCT04770220) is ongoing. It intends to enroll 300 APOE4 homozygotes with early to mild AD who receive 265 mg of ALZ-801 or placebo twice daily for 18 months. To date, data from these two studies has not been published. Depending on the progress of the studies, Alzheon decides to submit its marketing application to the FDA in 2025.

#### Gantenerumab

Gantenerumab is a purely human IgG1 antibody developed by Roche that targets Aβ fibrils. Its primary study population consists of patients with early AD of all genotypes. Its chemical structure remains a mystery. The therapeutic rationale for this antibody is that it eliminates amyloid plaques *via* Fcy receptor-mediated microglial phagocytosis and binds with high affinity to aggregated Aβ-species (21–23). *In vivo*, gantenerumab suppresses oligomeric Aβ42's neurotoxic effects (23). The PK profile of gantenerumab after subcutaneous administration was shown to attain a peak plasma concentration at a median time of 119h (about 5 days) and then fell in a mono-exponential way in a Phase I study (NCT02882009) (24). It showed that subcutaneous gantenerumab injections at rates of 5 and 15 s were well-tolerated in healthy volunteers, potentially allowing patients with AD or their caretakers to administer the drug at home (24). Aβ plaque levels were below the Aβ positive criterion in 37 and 51% of patients at years 1 and 2, respectively, in a positron emission tomography (PET) sub-study interim analysis of Roche's Scarlet RoAD (NCT01224106) and Marguerite RoAD (NCT02051608) (25). At 2 years, 51% patients receiving up to 1,200 mg of gantenerumab had PET amyloid levels consistent with sparse to non-neuritic Aβ (25). In 2021, the FDA designated gantenerumab as a breakthrough therapy. According to the favorable procedure of gantenerumab, Roche intends to submit a new drug application (NDA) in 2022.

#### Lecanemab

Biogen and Eisal collaborated on the development of lecanemab, formerly known as BAN2401. The target population of lecanemab is early AD patients of all genotypes and APOE4 carriers (10). There is no information available online about the chemical structure of lecanemab. Lecanemab is an antibody that binds to a soluble, poisonous form of Aβ (26). The binding is intended to neutralize Aβ and “tag” them so that the immune system can clear them from the brain before they aggregate and become plaques (26). The humanized IgG1 variant of the mouse monoclonal antibody mAb158 binds preferentially to soluble monomeric forms of Aβ. MAb158 has been shown to reduce Aβ protofibrils in the brain (42% less) and CSF in tg-ArcSwe mice (53% less) (27). In addition, mAb158 was found to protect neurons and reduce the toxicity of Aβ-protofibrils in neuron-glia co-cultures of mice by counteracting the abnormal accumulation of these protofibrils in astrocytes (28). Compared with the anti-amyloid antibodies aducanumab and gantenerumab, lecanemab was found to bind most strongly to Aβ-protofibrils, preferring the other highly aggregated forms (29). The sponsor conducted a combined single- and multiple-ascending dose study (NCT01767311) in August 2010 to evaluate the safety, tolerability, immunogenicity, pharmacodynamic response, and pharmacology of intravenous lecanemab in participants with mild to moderate AD. The incidence of ARIA-E/H on magnetic resonance imaging (MRI) was comparable to that of the placebo group. In all cases, lecanemab was extremely safe (30). However, the 12-month primary endpoint was not met (31). According to Bayesian and frequentist analyses, lecanemab at a dose of 10 mg/kg every 2 weeks reduced brain amyloid (0.306 SUVr units) and showed a difference between drug and placebo in favor of active treatment by 27 and 30% on the Alzheimer's Disease Composite Score (ADCOMS), 56 and 50% on the 14-item Alzheimer's Disease Assessment Scale, Cognitive Subscale (ADAS-Cog14), and 33 and 26% on the Clinical Dementia Rating Scale-Sum of Boxes (CDR-SB) compared with placebo at 18 months (31). Biomarkers in the CSF confirmed the treatment effect (31). Eisal initiated a phase III study called Clarity AD (NCT03887455) in March 2019 to evaluate the safety and efficacy of lecanemab in people with early AD. The Alzheimer's Clinical Trial Consortium (ACTC) launched a large trial (NCT04468659) next year that is expected to last until 2027 and was co-funded by the National Institutes of Health (NIH) and Eisal. The FDA designated lecanemab as a breakthrough therapy in June 2021 and granted it fast-track designation in late 2021 to expedite its review. The sponsors submitted the NDA to the FDA for approval in September 2021.

#### Donanemab

Lilly is exploring donanemab, a biologic that binds to deposited amyloid plaques in the brain, for the treatment of early AD in all genotypes (10). The chemical structure of donanemab has not yet been published. Donanemab targets an N-terminal pyroglutamate Aβ epitope found only in existing plaques (32–34). It is epitope specific, with no off-target binding to other Aβ species, neurotransmitters, or their receptors, and no known symptomatic effect (35). The MOA of donanemab is not just about preventing plaque deposition or growth, but rather about targeting deposited plaques to eliminate the existing amyloid burden in the brain. Some previous plaque-binding antibodies have been withdrawn because they caused microbleeds in the brain (36). Donanemab was well-tolerated in a dose-escalation study up to 10 mg/kg. And even 10 mg/kg caused significant changes in amyloid deposition (40–50%). It was observed that increasing the dose prolonged the mean terminal elimination half-life (34). Lilly undertook TRAILBLAZER-ALZ, a Phase II trial (NCT03367403) in early symptomatic AD to evaluate the safety, tolerability, and efficacy of donanemab. Reductions in amyloid plaques and global tau burden with donanemab were 85.06 centiloids and 0.01 greater than with placebo, respectively, at 76 weeks (35). Despite mixed secondary outcomes, donanemab has a better composite score for cognition and ability to perform activities of daily living than placebo (35). In two phase II studies, both infusion reactions occurred. Lilly began recruiting in TRAILBLAZER-ALZ 2 (NCT04437511), a phase II safety and efficacy study, in October 2020. It was then expanded to a Phase III registration study with 1,500 participants, with results expected in the first half of 2023 (36). Lilly is currently conducting three Phase III studies: TRAILBLAZER-EXT (NCT04640077), TRAILBLAZER-ALZ 3 (NCT05026866) and TRAILBLAZER-ALZ 4 (NCT05108922). In June 2021, the FDA granted donanemab breakthrough therapy designation to accelerate its development. Lilly submitted a regulatory filing in October 2021, with study data being submitted on a rolling basis.

#### ALZT-OP1

ALZT-OP1 is a combination product developed by AZ Therapies that includes a dry powder inhaler with cromolyn (ALZT-OP1a) and an oral tablet containing ibuprofen (ALZT-OP1b) (Figure 2B) (37). The target population is mild AD APOE4/4 homozygotes (10). Ibuprofen, a non-steroidal anti-inflammatory drug (NSAID) that is a cyclooxygenase (COX)-1 and COX-2 inhibitor and an agonist of peroxisome proliferator-activated receptors (PPARs), reduces nitric oxide (NO) synthesis, protects neurons from glutamate toxicity, and decreases production of pro-inflammatory cytokines. Ibuprofen penetrates the blood-brain barrier (BBB) and decreases neuritic plaque pathology and inflammation in the brains of people with AD. Ibuprofen is also a potent free radical scavenger that may help minimize lipid peroxidation and free radical formation. Because of its neuroprotective potential, relative safety, and long-standing use, ibuprofen has the potential to treat AD (38). Cromolyn sodium has been shown to effectively disrupt Aβ aggregation *in vitro* and dramatically reduce the amount of soluble Aβ *in vivo* after 1 week. Cromolyn sodium, alone or in combination with ibuprofen, significantly reduced aggregated Aβ levels and induced a neuroprotective microbial activation state favoring Aβ phagocytosis vs. a pro-neuroinflammatory state in APP^Swedish^-expressing Tg2576 mice (39). In a Phase I plasma/CSF PK crossover trial (NCT02482324) conducted in June and July 2015 with 26 healthy volunteers at Panax Clinical Research in Florida, AZ Therapies evaluated two ALZT-OP1 dosing regimens, each lasting 2 days. The combination was found to be safe; three patients reported mild to moderate AEs (37). Cromolyn and ibuprofen levels in CSF are thought to be sufficient to titrate the estimated daily amyloid production of 17.7 ng and the associated inflammatory response (40). In September 2015, ALZT-OP1 entered phase III. The study (NCT02547818) which evaluated the safety and efficacy of ALZT-OP1 in subjects with early AD, was completed in November 2020. Further information remains to be seen.

#### Semaglutide

**Figure 3:**
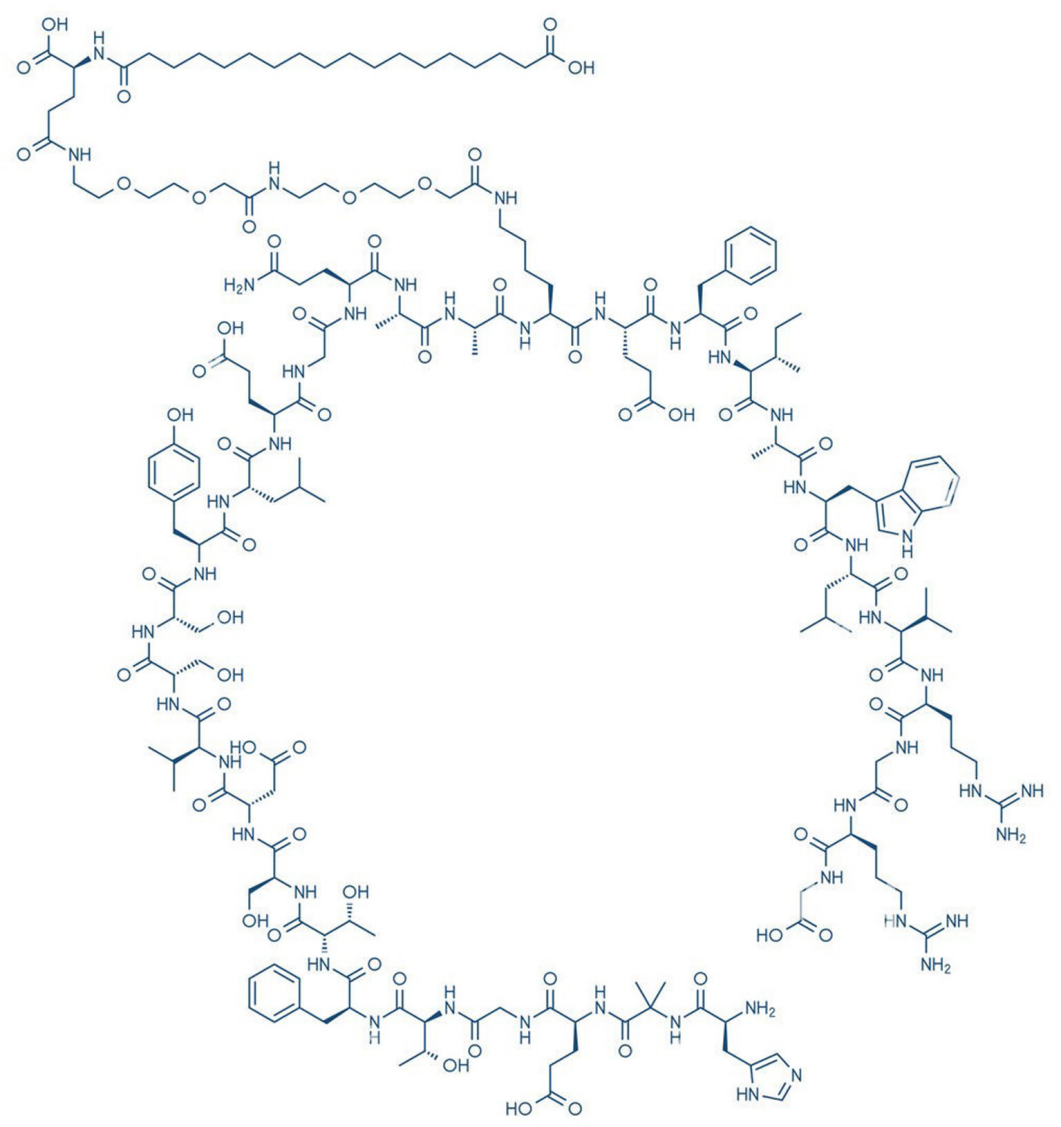
Chemical structure of semaglutide.

Semaglutide is being explored to improve insulin resistance in the treatment of AD. Semaglutide, an approved anti-diabetic drug, is a synthetic, long-acting version of glucagon-like peptide-1 (GLP-1) (Figure 3). GLP-1 is a hormone produced in the gut that stimulates the release of insulin and improves insulin sensitivity by activating receptors in the pancreas, liver, and gut (41). GLP-1 has the ability to cross the BBB and may improve insulin signaling in the brain (42, 43). It also enhances synaptic plasticity, cognition, and cell survival in the hippocampus nucleus (44). GLP-1 can be engineered to penetrate the BBB and exhibit neuroprotective properties *via* lowering inflammation, oxidative stress, and other factors (42). Semaglutide has been shown in preclinical studies to increase LC3II, Beclin-1, and P62, all of which block Aβ25-35 and hence accelerate autophagy. It also prevents apoptosis by reducing Bax expression caused by Aβ25 and enhancing Bcl2 expression decreased by Aβ25-35 (45). The Alzheimer's Association's Part the Cloud program supported a Phase II trial, yet it has not been registered. Novo Nordisk ran a Phase IIIa trial in December 2020 for people with early AD. When compared to the placebo group, the semaglutide group had a 53% lower risk of acquiring dementia. In March 2021, the sponsor registered two Phase 3 trials, EVOKE (NCT04777396) and EVOKE Plus (NCT04777409), to evaluate the efficacy and safety of semaglutide in the treatment of early AD. Both will last till April 20, 2026.

### Anti-tau strategies

Tau proteins currently represent one of most promising targets to treat AD. Compared to anti-Aβ therapeutic strategies with a long history of equivocal results and minimal therapeutic benefit, anti-tau therapeutic strategies emerged relatively late and showed the potential for better therapeutic effect. However, candidates that target only tau are still at an early stage of development, so they have not been included in this review.

### Anti-amyloid and tau strategies

#### Simufilam

Simufilam, formerly PTI-125, is a unique small-molecule (oral) agent that represents a completely novel method for treating AD (Figure 2C). It returns altered filamin A (FLNA), a brain scaffolding protein, back to its original structure and function. FLNA is a scaffolding protein that modulates the actin cytoskeleton (46). It is required for the normal folding and function of Aβ and tau. FLNA in its altered form hinders the Aβ and tau proteins from folding properly. This leads them to cluster together in the brain, forming plaques and tangles. Simufilam binds to FLNA and attempts to restore its activity, which may alleviate disease symptom (47). In 2017, Cassava Sciences launched a Phase I safety study (NCT03784300) in which 24 healthy adults were given 50, 100, or 200 mg of simufilam (46). In early 2019, the company conducted a NIH-funded Phase IIa trial (NCT03748706) in people with mild to severe AD. The results showed total tau, neurogranin, and the light chain of the neurofilament all dropped by 20, 32, and 22%, respectively (48). P-tau (pT181) was diminished by 34% (48). Biomarkers of neuroinflammation in the CSF (YKL-40 and inflammatory cytokines) dropped by 5–14%, and the outcomes were similar in plasma (48). Aβ42 increased somewhat, which is a nice sign because a low Aβ42 level implies AD (48). According to the findings, simufilam prevents disease and slows neurodegeneration. The organization executed an NIH-funded Phase IIb study (NCT04079803) at 10 sites across the US from September 2019 to March 2020. The firm registered two Phase III trials (NCT04994483 and NCT05026177) in the fall of 2021. However, Cassava's trial data has been extensively attacked, the clinical trials' scientific integrity has been called into question, and some have even urged them to be halted immediately. Cassava has currently reached an agreement with the FDA to conduct the Phase III trial. Cassava stated as recently as the beginning of October that it had begun the analysis of the late-stage project plan.

#### NE3107

NE3107 (previously HE 3286) is an anti-inflammatory, blood-brain permeable small molecule insulin sensitizer that binds to extracellular regulated protein kinases (ERK) (Figure 2D) (49). It has been demonstrated to reduce inflammation-driven ERK- and nuclear factor kappa-B (NF-κB)-stimulated inflammatory mediators, including tumor necrosis factor (TNF-α), without interfering with their homeostatic functions (50). NE3107 inhibits inflammation and improves insulin sensitivity, all of which have been proven to diminish AD pathogenesis in preclinical and clinical trials. It reduces inflammation by blocking EPIC, which is located upstream of Aβ and p-tau. It has no known interactions with nuclear hormone receptors, passes the BBB, has a high safety profile, and no indication of immunosuppression (50). The goal of an ongoing pivotal phase 3 trial in mild and moderate AD (NCT04669028) that began in May 2021 is to see if NE3107's impact on inflammation and insulin resistance will help slow the rate of cognitive loss. The trial is now enrolling patients and expanding to 45 sites. The data readout is scheduled for mid-2023.

#### GV-971

GV-971, also known as sodium oligomannate, is an oligosaccharide combination derived from the marine algae Ecklonia kurome that is used in China to treat AD (Figure 2E) (51). The agent suppressed neuroinflammation by significantly modifying gut microbiota composition and modulating amino acid metabolism (including a significant reduction in phenylalanine and isoleucine levels) (51). As a result, sodium oligomannate impaired the relationship between brain Th1 cells and gut microbiota changes (51). In sodium oligomannate-treated mice, there was a reduction in brain Th1 cells, microglial activation, and various brain cytokines, as well as a reduction in Aβ plaque aggregation and tau phosphorylation, as well as an improvement in discrimination learning (52). The MOA by which GV-971 may act is yet to be defined. GV-971 has a limited oral bioavailability, and food has little influence on absorption of this compound (53). There are no human studies on GV-971 metabolites, and the excretion pathways are unknown (51). GV-971 PK has not been examined in certain groups, such as individuals with hepatic or renal impairment (53). Green Valley began a Phase III trial (NCT02293915) at 34 sites in China in April 2014. At 4, 12, 24, and 36 weeks, the treatment group outperformed the placebo group in terms of Alzheimer's disease Assessment Scale–Cognitive Subscale (ADAS-Cog) scores, as reported at Clinical Trials on Alzheimer's disease (CTAD) 2018. The Canadian International Business Immigration Consultant (CIBIC) showed a trend toward improvement, but no other secondary outcomes changed (54). A subgroup analysis revealed that drug effects were larger in those with lower Mini–Mental State Examination (MMSE) scores at the start of the study. GV-971 was well-tolerated. The experiment was completed by 80% of participants, and the rates of AEs were comparable in the treatment and placebo groups. The results of a 36-week experiment revealed that GV-971 displayed high efficacy in improving cognition, with persistent improvement throughout all observation periods (55). In November 2019, GV-971 gained its first approval in China for the treatment of mild to severe AD to improve cognitive function (51).

All the Disease-modifying therapy medications cited in this article have been listed in Table 1.

**Table 1:** Disease-modifying therapy in active development from 2017 to 2021.

**Candidate**	**Target**	**MOA**	**Sponsor**	**Phase globally**	**Phase in China**	**Last completed trial (Phase)**	**Patient enrolled**	**Disease stage**	**Reduce Aβ or/and tau burden**
Aducanumab	Aβ	Remove aggregated forms of Aβ	Biogen; Eisai	Approved	N	NCT02484547 (III)	1,643	Early AD	+
						NCT02477800 (III)	1,653		–
ALZ-801	Aβ	Prevent Aβ42 from forming oligomers	Alzheon	III	N	NCT04157712 (I)	127	HV	N/A
						NCT04585347 (I)			
Gantenerumab	Aβ	Disassemble and degrade Aβ fibrils	Roche	III	III	NCT01224106 (III)	194	Prodromal AD	+
						NCT04623242 (III)	799	Mild AD	+
lecanemab	Aβ	Disassemble and degrade Aβ fibrils	Eli Lilly	III	III	NCT01767311 (II)	856	Early AD	+
Donanemab	Aβ	Remove Aβ plaques	Eli Lilly	III	N	NCT03367403 (II)	272	Early AD	±
ALZT-OP1	COX-1; PPARs	Promote microglia recruitment to plaques, and phagocytosis of Aβ deposits	AZ Therapies	III	N	NCT02547818 (III)	620	Early AD	Unknow
Semaglutide	GLP1 receptor	Accelerate autophagy of Aβ25-35	Novo Nordisk	III	III	/	/	/	/
Simufilam	FLNA	Restore of normally folded Aβ and tau	Cassava	III	N	NCT03748706 (II)	13	Mild-to-moderate	+
		proteins	Sciences			NCT04079803 (II)	64	AD	+
NE3107	NF-κB	Reduces inflammation by blocking EPIC, which is located upstream of Aβ and p-tau	BioVie	III	N	/	/	/	/
GV-971	N/A	De-aggregate Aβ and reduce tau hyperphosphorylation	Shanghai Green Valley Pharmaceutical	Approved	Approved	NCT02293915 (III)	818	Mild-to-moderate AD	N/A

N/A, Not applicable.

## Symptomatic therapy

### Symptomatic for agitation

#### AXS-05

**Figure 4:**
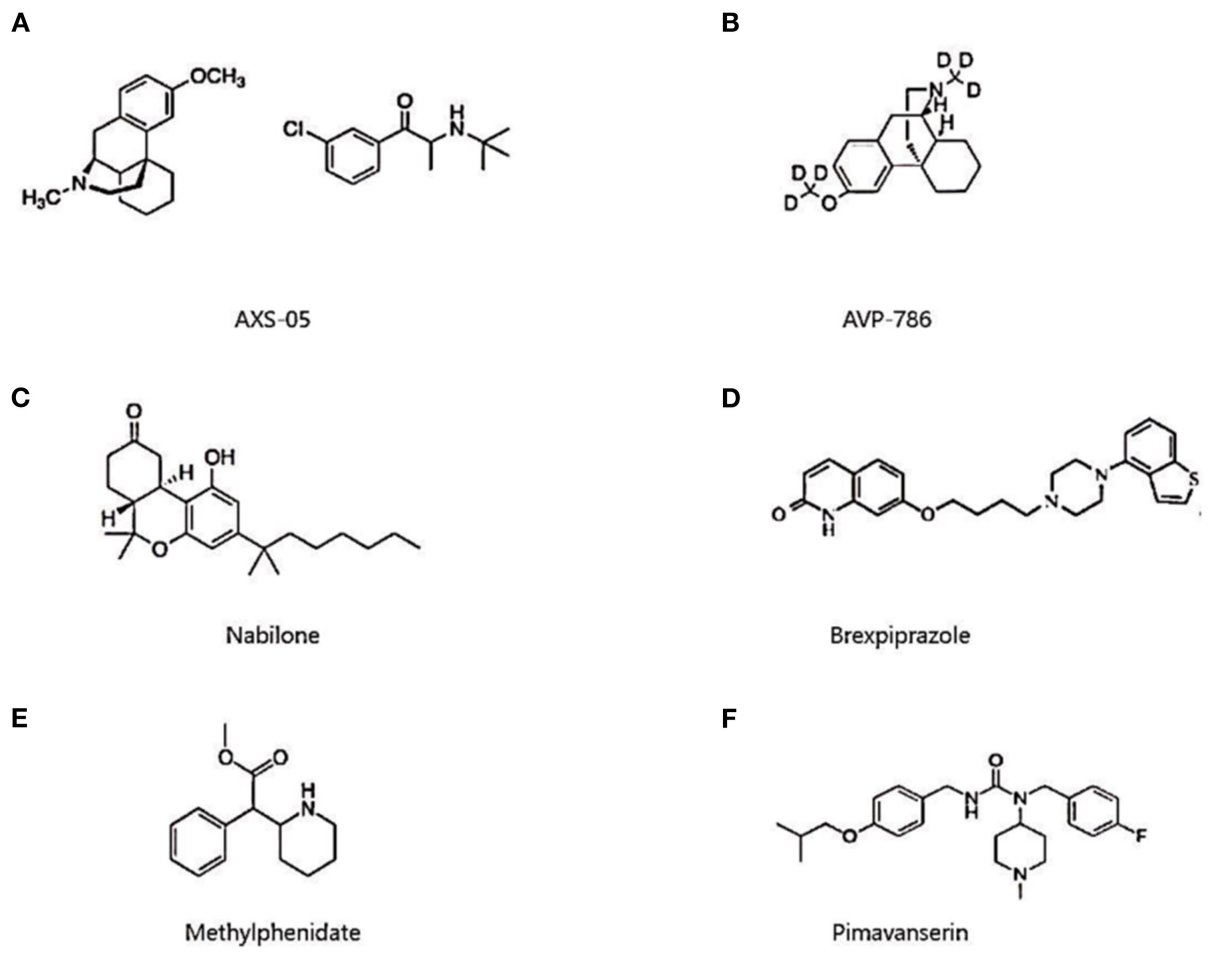
Chemical structure of AXS-05 **(A)**, AVP-786 **(B)**, nabilone **(C)**, brexpiprazole **(D)**, methylphenidate **(E)**, and pimavanserin **(F)**.

AXS-05 is an innovative, fixed-dose oral combination of two authorized medications being developed to treat agitation in AD (Figure 4A). Dextromethorphan (DM), one of the ingredients, is a weak antagonist of N-methyl-D-aspartate (NMDA) receptors, an agonist of sigma 1 receptors (endoplasmic reticulum membrane molecular chaperones), and an inhibitor of serotonin and norepinephrine transporters, nicotinic acetylcholine receptors, and microglial activation. Bupropion is another component whose major role is to increase the bioavailability of dextromethorphan by decreasing its metabolism and boosting its plasma levels while also reducing norepinephrine (NE) and dopamine reuptake (56). There is no information on Phase I trials in the trial register or the peer-reviewed literature. Axsome commenced a Phase II /III trial called ADVANCE-1 (NCT03226522) in July 2017 to evaluate the efficacy and safety of AXS-05 for the treatment of agitation in AD patients. The sponsor announced topline data in April 2020, citing a statistically significant 15.4 points reduction in Cohen-Mansfield Agitation Inventory (CMAI) with therapy, compared to 11.5 points with placebo and 10.0 points with bupropion alone. AXS-05 fulfilled the primary endpoint and resulted in a clinical response on the CMAI in over 70% of patients in this trial, defined as a 30% or higher improvement. It was also statistically significantly better than bupropion in terms of component contribution. AXS-05 was generally safe and well-tolerated, and was not associated with sedation-induced cognitive impairment (57). The FDA approved AXS-05 breakthrough therapy designation for treating agitation in AD in June 2020. Alongside that, Axsome announced that two trials to support the NDA for agitation in AD would begin before the end of 2020. The first will be a Phase III efficacy study (NCT04797715) while the second will be an open-label long-term safety study.

#### AVP-786

Avanir and Concert Pharmaceuticals created AVP-786, a deuterated second-generation version of AVP-923 (Figure 4B). It comprises DM and quinidine, two approved medicines. DM is the agent's neuroactive ingredient (58). Quinidine improves DM bioavailability by blocking the BBB protein pump P-glycoprotein and decreasing its oxidative metabolism by the liver enzyme cytochrome P450-2D6. AVP-923 is linked to less agitation in people with neurocognitive impairments, including AD (59). AVP-786 is distinct from AVP-923 in that it incorporates deuterium into the DM, which has been shown to reduce first-pass metabolism in the liver. Because of the lower metabolic rate, AVP-786 requires ultra-low quinidine doses, which improves overall safety and tolerability by reducing drug-drug interactions and adverse cardiac effects (60, 61). The safety, tolerability, and PK of DM dextromethorphan were evaluated in a phase I trial. At lower quinidine dosages, AVP-786 had the same steady-state plasma levels of DM as AVP-923. In November 2015, the FDA fast-tracked AVP-786 for agitation. The sponsor also ran two Phase III trials in the same year, TRIAD-1 and TRIAD-2 (NCT02442765 and NCT02442778), to analyze the drug in moderate AD patients with clinically significant agitation. Avanir revealed in March 2019 that the TRIAD-1 sequential design study had accomplished its primary endpoint, whereas TRIAD-2 had failed. There were no treatment-related deaths, although falls, urinary tract infections, headaches, and diarrhea were common AEs. The sponsors are currently undertaking the investigation on AVP-786 in AD agitation globally, with four trials registered on ClinicalTrials.gov (NCT04464564, NCT04408755, NCT03393520, and NCT02446132). In the future, positive outcomes are expected.

#### Nabilone

Cannabinoids (CB) have previously been shown to have a neuroprotective impact by activating the G-protein coupled receptors: CB1 and 2 (CB1/2) receptors (Figure 4C). CB1 receptors, which are found in the cerebral cortex and hippocampus, can impair learning and memory function in Alzheimer's patients and are also linked to anxiety-like and aggressive behavior in animals. CB2 receptors were engaged at the same time, reducing the generation of pro-inflammatory chemicals and removing Aβ plaques (62). Nabilone, a synthetic CB that acts as an agonist at CB1/2 receptors, has been studied to see if it improves agitation in AD. Sunnybrook Health Sciences Centre sponsored a Phase II/III trial (NCT02351882) in January 2015 to test the impact of 6 weeks of nabilone treatment on agitation symptoms compared to placebo (63). CMAI, Neuropsychiatric Inventory-Nursing Home version (NPI-NH) total, NPI-NH caregiver distress, and standardized MMSE were all favored by nabilone using a linear mixed model (64). Treatment differences favored placebo in those who completed the simultaneous integrated boost (SIB) (*n* = 25) (64). The difference in Certified Gastroenterology Coder (CGIC) improvement between nabilone (47%) and placebo (23%) was not statistically significant (McNemar's test, exact *p* = 0.09) (64). Sedation was higher during the nabilone (45%) vs. placebo (16%) phases (McNemar's test, exact *p* = 0.02), although treatment-limiting sedation was not substantially different (McNemar's test, exact *p* = 0.22), hence sedation and cognitive function must be continuously monitored (64). Following the completion of the study, the sponsor commenced a Phase 3 trial (NCT04516057) in February 2021. It will last until October 2025.

#### Brexpiprazole

Brexpiprazole is a unique third-generation antipsychotic that functions as a partial agonist for dopamine D2 receptors, a partial agonist for serotonin 5-HT1A receptors, an antagonist for serotonin 5-HT2A/5-HT2B receptors, and a partial antagonist for noradrenaline α1B/α2C receptors (Figure 4D) (65). Brexpiprazole may be beneficial in the treatment of dementia sufferers since serotonin, dopamine, and noradrenaline neurotransmitter systems are linked to behavioral symptoms of dementia, such as agitation (66). Otuska and Lundbeck collaborated in 2013 to conduct two 12-week Phase III studies (NCT01862640 and NCT01922258) to assess the efficacy, safety, and tolerability of brexpiprazole in patients with agitation associated with AD dementia. In the study (NCT01862640), brexpiprazole 2 mg and 1 mg significantly reduced total CMAI scores by 21.6 and 17.6 points, respectively, at 12 weeks, compared to 17.8 points in the placebo group, and greatly decreased NPI-NH agitation/aggression scores by 58.34 and 51.71%, respectively, at 12 weeks, compared to 47.12% for placebo (66). Brexpiprazole 0.5–2 mg/day did not establish statistical advantage over placebo in study (NCT01922258). In *post-hoc* studies, however, patients titrated to the maximal brexpiprazole dose of 2 mg/day indicated a benefit when compared to similarly titrated placebo patients (66). Brexpiprazole provoked treatment-emergent adverse events (TEAEs) in both studies, including headache, insomnia, urinary tract infection, and others. The bulk of the TEAEs were mild to moderate in severity (66). In 2018, the sponsors commenced three Phase III trials l (NCT03594123, NCT03548584, and NCT03724942) and one Phase II/III trial (NCT03620981), all of which are scheduled to run through 2022.

### Symptomatic for apathy

#### Methylphenidate

Methylphenidate has been approved for narcolepsy and attention deficit hyperactivity disorder (ADHD) (Figure 4E). Methylphenidate hinders presynaptic neurons from reabsorbing two neurotransmitters, adrenaline and dopamine. It inhibits the transporters of these neurotransmitters, resulting in an increase in dopamine and adrenaline levels in the synaptic cleft (67). It is also a mild agonist at the 5-HT1A receptor, which is another mechanism that contributes to higher dopamine levels (68). Methylphenidate can promote neuroprotection *via* increasing dopamine levels (69). Apathy in people with AD is thought to be caused by reduced dopaminergic neurotransmission (70). It is being investigated as a treatment for apathy in patients with AD as a mild CNS stimulant. Methylphenidate may benefit non-cognitive, apathy-like behavior (as demonstrated by decreased exploration) in the 5xFAD mouse model, but it has little ability to enhance chronic Alzheimer's typical learning and memory deficiencies (71). It was shown that methylphenidate was well-tolerated and alleviated apathy in patients with AD in two open-label studies (72, 73). Another placebo-controlled crossover trial found that, in comparison to placebo, methylphenidate patients' apathy was considerably improved (74). A Phase II study called ADMET (NCT01117181) was established in June 2010 to further verify methylphenidate's involvement in AD. Methylphenidate improved NPI apathy scores by 1.8 points more than placebo, and further findings showed that methylphenidate reduced apathy in Alzheimer's patients and enhanced global cognition with few AEs (75). Methylphenidate was then evaluated in Phase III (NCT02346201) in 2016 for its ability to minimize the severity of apathy in AD. The NPI apathy score in those receiving methylphenidate decreased more from baseline to 6 months than in those taking placebo, and its safety was clearly proven in this experiment (76).

### Symptomatic for other psychosis

#### Pimavanserin

Pimavanserin has been approved for the treatment of Parkinson's disease psychosis (PDP) (Figure 4F). It is a selective inverse agonist/antagonist of the 5-HT2A receptor with little affinity for the 5-HT2C receptor and no affinity for dopaminergic, muscarinic, histaminergic, or adrenergic receptors (77). Previous research revealed that activation at the 5-HT2A receptor could possibly help with AD psychosis (78). It is now being developed for the treatment of psychosis in Alzheimer's patients. A prior study found that serotonin signaling attenuated Aβ *in vitro* and in animal models of AD (79). Pimavanserin administration interfered with Aβ deposition by altering the balance between two 5-HT2A signaling pathways that were antagonists of Gq/11 signaling and agonists of Gαi1 signaling, according to the findings (80). From November 2013 through September 2019, Acadia launched a Phase II trial (NCT02035553). At the primary endpoint (week 6), pimavanserin had an effect on psychosis and was well-tolerated. Further follow-up through week 12 revealed no meaningful benefits for pimavanserin as compared to placebo (81). A subsequent subgroup analysis revealed that ≥30% improvement was 88.9 vs. 43.3% (*p* < 0.001) and ≥50% improvement was 77.8 vs. 43.3% (*p* = 0.008) for pimavanserin and placebo, respectively, in this severe subgroup with a baseline Neuropsychiatric Inventory-Nursing Home Version psychosis score (NPI-NH-PS) ≥12 (*n* = 27 pimavanserin; *n* = 30 placebo) (82). The next Phase II study, SERENE (NCT03118947), was terminated in February 2017. The findings of this experiment do not support pimavanserin's function. Pimavanserin entered Phase III from 2017 to 2019. The trial (NCT03325556) sought to examine the efficacy of pimavanserin vs. placebo in preventing the return of psychotic symptoms in dementia-related psychosis patients who responded to 12 weeks of open-label pimavanserin treatment. According to the findings, 13% of the pimavanserin group relapsed, compared to 28% of the placebo group. The trial was also halted due to ineffectiveness. Longer and larger trials are needed to confirm the efficacy of pimavanserin on psychosis in AD (83). The sponsors submitted an NDA to the FDA in December 2021 for permission to treat AD.

All the symptomatic therapy medications cited in this article have been cited in Table 2.

**Table 2:** Symptomatic therapy in active development from 2017 to 2021.

**Candidate**	**Target**	**MOA**	**Sponsor**	**Phase globally**	**Phase in China**	**Last completed trial (Phase)**	**Patient enrolled**	**Disease stage**	**Relieve symptoms**
AXS-05	NMDA receptor; Sigma 1 receptor	Inhibit serotonin and NA transporters, N acetylcholine receptors, and microglial activation	Axsome Therapeutics	III	N	NCT03226522 (II/III)	366	AD agitation	+
AVP-786	NMDA receptor; Sigma 1 receptor	Inhibit serotonin and NA transporters, N acetylcholine receptors, and microglial activation	Avanir Pharmaceuticals; Concert Pharmaceuticals; Otsuka Pharmaceutical	III	N	NCT02442765 (III) NCT02442778 (III)	410 522	AD agitation	+ –
Nabilone	CB1/2 receptor	Reduce the generation of pro-inflammatory chemicals; Remove Aβ plaques	Other Hospital/Academic/ Medical Center	III	N	NCT02351882 (II/III)	38	AD agitation	+
Brexpiprazole	D2 receptor; 5-HT1A receptor; 5-HT2A/B receptor; NAα1B/α2C	D2 and 5-HT1A receptor partial agonist; 5-HT2A/5-HT2B receptor antagonist; NAα1B/α2C partial antagonist	Lundbeck	III	N	NCT01862640 (III) NCT01922258 (III) NCT03724942 (III) NCT03548584 (III)	433 270 164 345	AD agitation	+ + Unknown Unknown
Methylphenidate	5-HT1A receptor; SLC6A2	Block NE and DA reuptake; 5-HT1A receptor agonist	Other Hospital/ Academic/Medical Center	IV	N	NCT02346201 (III)	200	AD apathy	+
Pimavanserin	5-HT2A receptor	5-HT2A receptor agonist	ACADIA Pharmaceuticals	III	N	NCT03325556 (III)	392	AD psychosis	+

## Conclusion and future expectations

Available drugs for AD are only symptomatic in China, and even in the world. Prior to 2017, the four approved drugs for AD were acetylcholinesterase (AChE) inhibitors and NMDA antagonists. By lowering AChE activity, these medications temporarily halt the loss of cognitive function, resulting in increased ACh levels and enhanced brain function (84). However, they only provide minor benefits in terms of symptom management and do not prevent neuronal death, brain shrinkage, and cognitive decline (85).

Due to a lack of powerful drugs to slow the progression of AD, huge efforts have been undertaken to find novel molecules with the potential to change the illness's course: disease-modifying therapy (DMT) (86). Since AD evolves concurrently with the accumulation of Aβ, causing the expansion of tau pathology (87), these prospective DMT are essentially addressing the two pathogenic hallmarks of AD: Aβ and tau-protein (86). The amyloid hypothesis has been the subject of contemporary study (86). Aβ is the most popular target in Phase III drug development programs (87). Gantenerumab, aducanumab, ALZ-801, ALZT-OP1, donanemab, lecanemab, and semaglutide are among the selected agents aimed at eliminating Aβ. They are engaged in secondary prevention trials in people with preclinical, prodromal, mild, or moderate-to-severe AD (87). The risk of AD is 60–80% heritable, with more than 40 AD-associated genetic risk loci already identified, the APOE alleles having the strongest association with the disease (87). AD patients with APOE4/4 homozygotes are the target population for ALZ-801 and ALZT-OP1. Gantenerumab, aducanumab, and donanemab are beneficial in all genotypes of AD patients. Lecanemab is distinct in that it is intended for patients of all genotypes, including APOE4/4 homozygotes (19). According to prior studies, insulin resistance has been linked to neurodegeneration (88). Semaglutide is an anti-diabetic medication that has been approved in China. A trial in China is now underway to treat MCI and mild dementia caused by AD. Other agents, such as aducanumab, which has been approved in the US and is advised for clinical therapy, have not been studied in China aside from gantenerumab, lecanemab, and semaglutide. China has been paying attention to drug R&D in this field in recent years, and these promising candidates are expected to be expanded to China for future research. Tau biology adds to the list of potentially relevant targets for DMT (89). The anti-tau strategies, on the other hand, are still in the early stages of clinical studies (86). However, there are candidates that target both Aβ and tau, such as simufilam, NE3107, and GV-971. FLNA is a unique target in that recovered FLNA can restore normal Aβ and tau function. According to the existing data, simufilam is rather effective in AD. Neuro-inflammation is recognized as a major component of the pathology of AD, contributing to disease progression and neurodegeneration (87), in addition to aberrant Aβ protein deposition and hyperphosphorylation of tau protein. Therefore, medications targeting this pathway are also being developed to alleviate the condition of AD patients. NE3107 is a candidate that significantly targets inflammation, and some other candidates have anti-inflammatory properties as well. In addition to its anti-inflammatory properties, NE3107 has the potential to reduce insulin resistance. However, NE3107 is presently only being tested in clinical studies in the US. GV-971 belongs to DMT as well, although its MOA is undetermined and may be related to gut flora. It was approved in China in 2019, following a phase III trial that indicated cognitive enhancement in the same country (52). However, due to the difficulties to get more clinical reports for GV-971 in order to assess the quality of the evidence, the current Chinese guidelines have been unable to make adequate recommendations, and we only hope to augment it when the guidelines are revised in the future (4). All the agents described above were devised to combat the pathogenic mechanism of AD and, therefore, can interfere with the disease process. And, in disorders like Alzheimer's, pathogenic alterations occur before symptoms manifest. If patients can be diagnosed early, these compounds could become the top choice for the prevention and treatment of AD, changing the existing status of no cure.

Cognitive impairment and psycho-behavioral dysfunction are the two primary categories of AD symptoms. The four approved Alzheimer's medications are generally used to modify cognitive impairment, and more are being developed to improve patients' cognition by delaying the disease process in the past 5 years. Antipsychotic behavioral disorder drug investigation accounts for a significant portion of AD symptomatic treatment. Pimavanserin is an inverse agonist for the 5-HT2A receptor that has been investigated for dementia-related psychosis (87). Only the treatment of hallucinations and delusions associated with psychosis in Parkinson's disease (PD) has been studied with pimavanserin in China. However, because pimavanserin relieves hallucinations and delusions while having no harmful effects on cognition, the Chinese guideline makes the following recommendation: Pimavanserin has a short-term effect on AD dementia mental symptoms (4). Apathy is one of the symptoms of depression, which is common in Alzheimer's patients. The guidelines show that tandospirone, but not sertraline or mirtazapine, is beneficial for depression in Alzheimer's patients (4). In recent research, methylphenidate has been considered to be an effective symptom-modifying drug (90). In China, methylphenidate is recommended for respiratory depression, narcolepsy, ADHD, and other conditions. Agitation is a prevalent symptom of dementia, affecting up to 70% of patients with AD during their illness (91). Some atypical antipsychotics (such as olanzapine, risperidone, and quetiapine, among others) and serotonin medications (such as pimavanserin, buspirone, and citalopram, among others) have been recorded in the guidelines for the effect of alleviating psychological symptoms, mainly agitation, in AD patients (4). Recent studies have demonstrated that AXS-05, AVP-786, nabilone, and brexpiprazole are significantly effective in treating agitation. Clinical studies for AXS-05 and AVP-786 are not being conducted in China. As nabilone is illegal in China, it cannot be used clinically. Brexpiprazole was initially developed for depression and schizophrenia and has not been linked to the agitation of AD in China. It's challenging to compare the efficacy of the above-mentioned drugs since the primary endpoints of clinical trials are inconsistent, but they've all shown curative effects, giving doctors more options to treat AD patients with psychiatric symptoms in the future.

In the last 5 years, the R&D pipeline for AD has been primarily focused on DMT, with the goal of improving the embarrassing status of no viable pharmacological treatment in clinic. Agents targeting Aβ have a distinct advantage over late-stage agents, despite the fact that many agents' development processes are tortuous. Tau protein, a new intriguing target, may be more effective than Aβ in treating AD, and the future pipeline may focus on this target. If the tau protein study continues to make significant progress, it will be a tremendous step forward in the treatment of AD. New targets for different pathogenic pathways, like FLNA and improving blood glucose, provide new opportunities for future R&D pipelines. In the R&D pipeline of AD, drugs that relieve symptoms still have a place. Although the number of potential pharmaceuticals developed in China is still limited, China is actively introducing and looking forward to participating in the development of these drugs. Once approved by the National Medical Products Administration (NMPA), these drugs will be able to remedy the shortages of radically curative drugs and provide more therapeutic alternatives for symptomatic therapy.

## Author contributions

YC searched the database and conducted analysis. YC and YL wrote original draft. YL and XL did review and editing and were responsible for supervision.

## Funding

This work was supported by Department Resource.

## Conflict of interest

The authors declare that the research was conducted in the absence of any commercial or financial relationships that could be construed as a potential conflict of interest.

## Publisher's note

All claims expressed in this article are solely those of the authors and do not necessarily represent those of their affiliated organizations, or those of the publisher, the editors and the reviewers. Any product that may be evaluated in this article, or claim that may be made by its manufacturer, is not guaranteed or endorsed by the publisher.
